# Quantifying drift-selection balance using an agent-based biofilm model of identical heterotrophs under low-nutrient conditions

**DOI:** 10.1098/rsfs.2023.0010

**Published:** 2023-06-09

**Authors:** Joseph Earl Weaver

**Affiliations:** School of Civil Engineering and Geosciences, Newcastle University, Cassie Building, Newcastle upon Tyne NE1 7RU, UK

**Keywords:** agent-based model, biofilm, drift, neutral assembly, community assembly, individual-based model

## Abstract

Both deterministic and stochastic forces shape biofilm communities, but the balance between those forces is variable. Quantifying the balance is both desirable and challenging. For example, drift-driven failure, a stochastic force, can be thought of as an organism experiencing ‘bad luck’ and manipulating ‘luck’ as a factor in real-world systems is difficult. We used an agent-based model to manipulate luck by controlling seed cevalues governing random number generation. We determined which organism among identical competitors experienced the greatest drift-driven failure, gave it a deterministic growth advantage and re-ran the simulation with the same seed. This enabled quantifying the growth advantage required to overcome drift, e.g. a 50% chance to thrive may require a 10–20% improved growth rate. Further, we found that crowding intensity affected that balance. At moderate spacings, there were wide ranges where neither drift nor selection dominated. Those ranges shrank at extreme spacings; close and loose crowding, respectively, favoured drift and selection. We explain how these results may partially illuminate two conundrums: the fact that a stably operating wastewater treatment plant's microbial community can vary greatly over time and the difference between equivalent and total community size in neutral community assembly models.

## 1. Introduction

Both stochastic and deterministic assembly processes can shape biofilm communities [1,2]. Those processes, however, rarely act equally and the balance between them is determined by many conditions related to competition intensity. Such conditions include population size [3,4], available space [5] and resource availability [6]. Understanding how this balance shifts under differing conditions provides insights into biofilm-associated systems such as environmental bioreactors, healthcare, industrial production and natural ecosystems.

Here, we attempt to quantify the balance between drift, a pure stochastic process [1,3] and a more deterministic kinetic advantage. Under this balance, even if losing the ‘drift lottery’, an individual's progeny may thrive if their maximum growth rate (*μ*_max_) or half-saturation constant (*K_s_*) confers a selection advantage over their competitors.

Such quantification is challenging. Drift is an inherently random process and experimental manipulation of a random process, distinct from simply controlling for it, is difficult. Despite that difficulty, there have been some physical experiments in which drift is isolated as an experimental factor [4,7,8], often requiring subtle statistical analyses or extremely precise experimental work.

An alternative approach, used here, is to perform the experiments *in silico* where drift may be directly manipulated via random number generation. We used an agent-based model (Newcastle University Frontiers in Engineering Biology (NUFEB)) [9,10] to simulate spatially competing bacteria under low-nutrient conditions. The bacteria were identical and evenly spaced, differentiated only by random growth directions and biomass allocations during division. Drift was therefore the only selection process and was controlled by the seed value initializing the random number generator.

Our goal was to determine the degree to which a deterministic factor (here, Monod kinetics) must improve to overcome drift-driven failure so subsequent simulations using identical seeds were run. The difference was that the ‘biggest loser’, the lineage with the lowest relative abundance, was assigned different kinetics. This approach allowed us to relate quantifiable kinetic changes to the likelihood that the failing lineage would overcome drift-driven failure and thrive. We also determined how the required degree of change varied under differing crowding intensities (e.g. closer spacing and increased initial population size).

We found that under purely stochastic conditions the losing lineage varied unpredictably between runs, showing the expected effects of drift. Further, altering kinetics did enable losing lineages to overcome drift. For example, for an initial population of nine cells evenly spaced 10 diameters apart either *K_s_* or *μ*_max_ had to improve by at least 10–20% for a 50% chance of thriving. Crowding affected both the improvement needed for a 50% chance of thriving and the ranges over which both drift and fitness influenced success. The strong and sometimes nonlinear interactions between terms could not be adequately reproduced using simple linear estimators but could be adequately expressed with a generalized additive model (GAM).

## 2. Methods

### 2.1. Agent-based model

The agent-based model employed NUFEB [9,10], which is based on the LAMMPS [9] molecular dynamics simulation framework and has successfully been used to model multi-species biofilms [10], including development and detachment [7], trade-offs in extracellular polymeric substance production [11], and phototroph–heterotroph metabolic interactions [12].

NUFEB is not lattice based; cells were positioned in three dimensions and had individual dynamic sizes. The directions in which cells divided and biomass allocations (40 to 60%) during division were randomly determined using a Park–Miller pseudorandom number generator and were the two factors contributing to drift.

The individually simulated bacterial cells physically interacted using realistic physics and grew according to Monod-style models described by equation (2.1) where *µ* is the substrate-dependent growth rate (1/h), *µ*_max_ is the maximum specific growth rate (1/h), [*S*] is the concentration of the relevant substrate (kg m^−3^) and *K_s_* is the half-saturation constant for the substrate (kg m^−3^). Additional descriptions of NUFEBs mechanics are detailed in previous publications [9,10].

2.1 $$\mu =\;\mu_{\max}\frac{\left[S\right]}{K_{s}+[S]}\;.$$

The simulation volume height (2 × 10^−4^ m) was defined to be in the Z-dimension, the bulk substrate concentration boundary condition at the top of the simulation volume was 1 × 10^−4^ kg m^−3^ and the initial substrate concentration throughout the volume was set to the same value. The X and Y dimensions were equal and varied based on spacing and number of initial cells. Additionally, the X and Y boundaries were periodic, allowing biomass and substrates to wrap from one side of the simulation to the other.

#### 2.1.1. Model implementation details

NUFEB simulates bacterial growth, physical interactions and substrate diffusion and reactions within a cuboid volume. Bacterial growth is given as mass over time and determined by a summation of Monod-style rate equations and the change in mass is used to calculate the diameter of a spherical organism. When an individual grows beyond a user-defined threshold (here 1.36 μm), it divides into two organisms. The first cell receives 40–60% of the biomass (uniformly randomly selected) and the second cell receives the remainder. The three-dimensional direction of division relative to the centre of the initial cell is randomly chosen. Mechanically, the individuals are subjected to contact, adhesion and fluid forces which are implemented as respective as spring and dashpot, spring and simple one-way coupling physical models. A mechanical relaxation step is performed to address the mechanical in-equilibrium introduced by organism division. With respect to the crowding explored in this research, the result of mechanical relaxation is that a freshly cell which finds itself ‘overlapping’ with existing biomass will be part of a ‘shoving’ match in which all relevant individuals will be pushed into nearby empty space.

In this simulation, a generic nutrient substrate is modelled and oxygen is non-limiting. The substrate is modelled within the cuboid by solving a standard advection–diffusion–reaction equation. The equation is discretized across and solved for small voxel subsections of the cuboid with a short time step.

The implementation used here does not differ from previous detailed explanations [10] employing thee the ODD protocol (Overview, Design concepts, Details), which is a standard for agent-based model description. Specifically, the underlying equations regarding growth, transport and physical interactions have not been modified, and the interested reader is guided specifically to the supporting information of [10] for an exhaustive, canonical description.

### 2.2. Experimental approach

The base experimental unit was an agent-based simulation initially seeded with identical bacterial cells with starting diameters of 1 × 10^−6^ m, *K_s_* of 3.5 × 10^−5^ kg m^−3^, *µ*_max_ of 1 h^−1^, and yield 0.61 kg biomass per kg substrate consumed. The initial cells (total population 4, 9 or 16) were arranged along evenly spaced (2.5, 5 or 10 cell diameters) *MxM* points at the base of the simulation volume. Bacteria were allowed to grow and compete until 20% of the simulation volume consisted of heterotrophic biomass.

Each combination of populations sizes and spacings was run 120 times using different seed values to initialize the random number generator and the ‘biggest loser’ from each run was identified (see §2.3). Those simulations were then run again, but with the failed lineage given altered kinetic values (see §2.4). The results of the runs were used to determine how the altered kinetics contributed to the probability of transitioning from drift-driven failure to a thriving state (see §2.5) under various crowding intensities.

All combinations of the factor levels listed in table 1 (1089 combinations) were simulated for each of the 120 seeds, resulting in a total of 130 680 runs. Each run required between 2 to 36 h to complete, so the simulations were carried out on a high-performance computing cluster (see §2.6).

**Table 1.** Experimental factors and levels.

factor	values										
spacing (cell diameters)	2.5	5	10								
initial population size	4	9	16								
percentage change in *K_s_*	−50	−40	−30	−20	−10	0	10	20	30	40	50
percentage change in *µ*_max_	−50	−40	−30	−20	−10	0	10	20	30	40	50

### 2.3. Determining failed lineages

For a system initialized with *N* bacterial lineages, the total biomass *X_t_* is the sum of the biomass for each lineage *X_i_,* as expressed by equation (2.2).

2.2 $$X_{t}=\overset{N}{\underset{i}{\sum }}X_{i}.$$

In a system where each initial cell is identical, with no competition and with no random effects, all *X_i_* are expected to be equal, thus the expected relevant abundance of any lineage (*X_E_*) is given as

2.3 $$X_{E}=X_{T}/N.$$

In the first round of simulations, all initial cells were identical and evenly spaced, but cell division directions and biomass allocations during division were determined randomly. As a result, the distribution biomass for any lineage at any particular time was often not equal to the expected relevant abundance, $X_{i}\neq X_{E}$. In practice, there were often one or two lineages which strongly dominated with *X_i_* ≫ *X_E_*, one or two lineages which became vanishingly small with *X_i_* ≪ *X_E_* (the ‘biggest losers’), and the rest persisted at some noticeable abundance that was however below *X_E_*. Moreover, the outcomes appeared to be determined early in the simulation, especially for the best- and worst-performing lineages (electronic supplementary material, figure S1, table S1 and video SV1). We have defined three classifications of lineage survival based on the difference between *X_E_* and *X_i_*: *languishing* $\left(X_{i}<0.3\;X_{E}\right)$, *thriving* $\left(X_{i}>0.9X_{E}\right)$ and *barely surviving* $\left(0.3\;X_{E}\leq X_{i}\leq 0.9X_{E}\right)$. The threshold for thriving is lower than XE to accommodate situations where single lineage massively dominated (e.g. *Xi* > 0.6) leading to lineages which were clearly otherwise doing well but with low relative abundance.

### 2.4. Kinetic alteration for potential selective advantage

**Figure 1.**
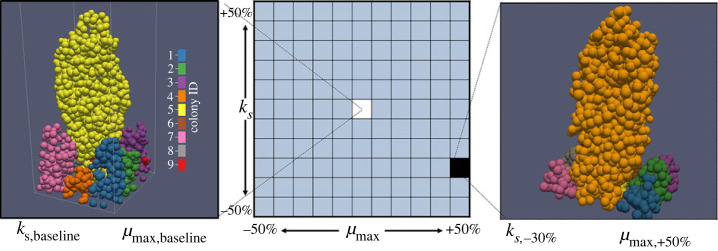
Illustration of a parameter sweep. Under baseline conditions when all bacteria are identical (left-hand side), colony 4 was the worst-performing lineage. When colony 4 was given a potential selective advantage (right-hand side) via reduced *K_S_* and increased *µ*_max_, colony 4 transitioned to thriving. This result along with all other parameter combinations across 120 random seeds was used to estimate *p*_thrive_, the probability that the worst-performing colony would transition to thriving under given altered kinetics. The trend of upward growth by the bacteria is due to substrate concentration gradients and is characteristic of growth under low-nutrient conditions [10].

The worst-performing bacterial lineages from each of the initial homogeneous runs were modified by altering their individual maximum specific growth rate (*µ*_max_) and/or their half-saturation constant (*K_s_*) (figure 1), potentially giving them a competitive advantage. The altered values were selected as described in table 1. We acknowledge that not all combinations of *µ*_max_ and *K_s_* were advantageous and that *µ*_max_ and *K_s_* are often strongly correlated; here our goal was to thoroughly explore the parameter space.

A two-dimensional parameter space was chosen because both *µ*_max_ and *K_s_* met two desirable criteria. First, they directly associate growth and substrate concentration. Second, they are major parameters used when designing bioreactors, calibrating associated models, and when discussing kinetic control of microbial populations within reactors. A composite ratio of the parameters did not appear usable due to a lack of symmetry in results (e.g. across the upper-left to lower-right diagonals in figure 4). The disadvantage of such an approach is the large computational cost. For similar work where those criteria do not apply, a one-dimensional parameter space is suggested. Ideally, this single-parameter would be part of the underlying biological model (such as yield), rather than a generic multiplicative ‘selective advantage’ variable.

### 2.5. Probability map generation

The kinetic parameter sweeps were used to generate tables for each combination of factors which listed the final relative biomass of each bacterial lineage, that lineage's status as the ‘biggest loser’, and the lineage's success under each run. Within each combination population size and spacing, the percentage of failing lineages which transitioned to thriving during the parameter sweep was recorded across all seeds. Those percentages represent the probabilities that the selective advantage (if any) conferred by altered kinetics would outweigh drift-driven failure under the given conditions.

### 2.6. Simulation management

Simulations were run and their results tabulated on the Newcastle University Rocket High Performance Computing environment and managed using Snakemake [13,14] workflows populating a SLURM [15] queue. Each simulation was run on a single core, with multiple hundreds of simulations run in parallel. Job submissions encompassed all kinetic parameter sweeps for each combination of other parameters, e.g. a single batch submission would consist of all combinations of *µ*_max_ and *K_s_* for four bacteria, spaced five diameters apart.

### 2.7. Data analysis

Simulation results were saved as tabular comma-separated value text files and aggregated using BASH [16] (v. 4.2) shell and Python [17] (v. 3.8) scripts which included the NumPy [18] and pandas [19] libraries. Further processing of the data was performed off the cluster and used R [20] (v. 4.2) scripts incorporating various Tidyverse [21] and other supporting packages [22–43].

#### 2.7.1. Parameters quantifying the balance between drift and selection

**Figure 2.**
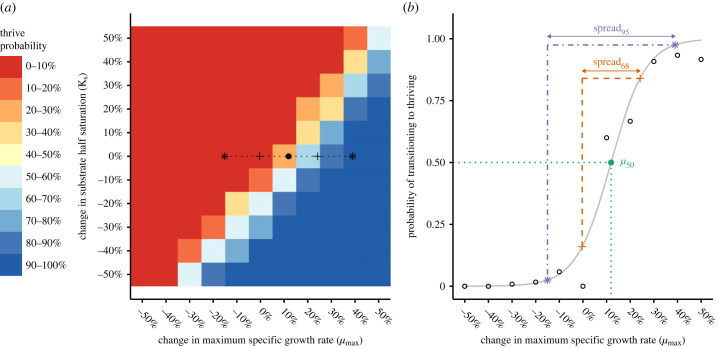
Illustration of how the *µ_50_* and *spread* parameters were calculated. In this example, the probability map corresponding to four initial organisms placed five diameters apart is shown (*a*), and the dashed line is drawn along a line of constant *K_s_*. The full length of the line denotes the *spread_95_* region, the portion between crosses denotes *spread_68_* and the solid point represents the *µ_50_* mark. When the *p*_thrive_ values are plotted as a function of *µ*_max_ along the line of constant *K_s_*, (*b*) it is apparent that a logistic function (grey solid line) may be fitted to the points (black rings). The fitted function was used to estimate both the value of *µ* corresponding to *µ_50_* and the widths of the *spread* regions. This analysis was repeated for all crowding conditions along all lines of constant *K_s_*.

Each probability map was conceptually analogous to a cliffside; a continuous sharp probability threshold gradient separated by two flat regions of either 100% lineage success or failure (figure 2*a*). We wished to quantify the midpoint and steepness of the gradient along lines of constant *K_s_* for each crowding condition. A cross-section of the probabilities along *µ*_max_ for any constant *K_s_* produces a sigmoid-shaped profile (figure 2*b*). The profiles were fit to a logistic function of *µ*_max_ with a maximum value of 1 given by equation (2.4), where *p*_thrive_ is the probability of transitioning to a thriving colony, *k* is a parameter affecting the steepness of the curve, and *µ_50_* is the *µ*_max_ value at which there is a 50% probability of thriving.

2.4 $$p_{\mathrm{thrive}}=\;\frac{1}{1+e^{-k*(\mu_{50}-\mu_{\max})}}.$$

The relevant *k* and *µ_50_* parameters from each fit were recorded. We also determined the domains of *µ*_max_ values associated with the $p_{\mathrm{thrive}}$ ranges covering either a 2.5–97.5% or 16–84% chance of thriving. These domains, respectively, named *spread_95_* and *spread_68_* quantified the regions over which both drift and selection influenced success.

The results of all sigmoid fits are shown in the electronic supplementary material, figures S2–S10.

#### 2.7.2. Analysing balance parameters

Within each crowding scenario, the extracted parameters were analysed using simple linear regression models of the parameters as functions of *K_s_.* The effect of crowding pressure (spacing and total population) was then analysed by comparing the results of the fits between scenarios.

We note that although the linear fits for a second-order polynomial on *µ_50_* generally resulted in marginally improved *R*^2^ scores and removed parabolic patterns from the residuals, the simple linear regressions were still excellent and more interpretable; care should be taken if extending this work to larger ranges of kinetic values.

#### 2.7.3. Modelling the effect of competitive pressure and altered kinetics

We wished to determine if a model based on the simulation results could accurately reproduce the transition probabilities for each crowding scenario. The ultimate goal of these models was not prediction, but to provide a descriptive framework [44] showing which factors, interactions and potential nonlinearities were important. Variations on both multiple linear regression (MLR) models and GAMs [45] were fitted to either the log-likelihood of *p*_thrive_ (for MLRs) or directly to *p*_thrive_ (GAMs).

In both cases, backward step selection from factorial models incorporating up to three-way interactions was performed to select the final model. Non-significant (*p* > 0.05) terms were iteratively removed from the model starting with the highest order interactions. Main effects were retained even if non-significant when they were part of a significant interaction term.

The final models were selected based on *R*^2^ and Akaike information criterion values as well as interpretability. The potential models and the associated fit criteria are included in the electronic supplementary material, tables S2–S5.

## 3. Results

### 3.1. Drift occurred when all cells were identical

**Figure 3.**
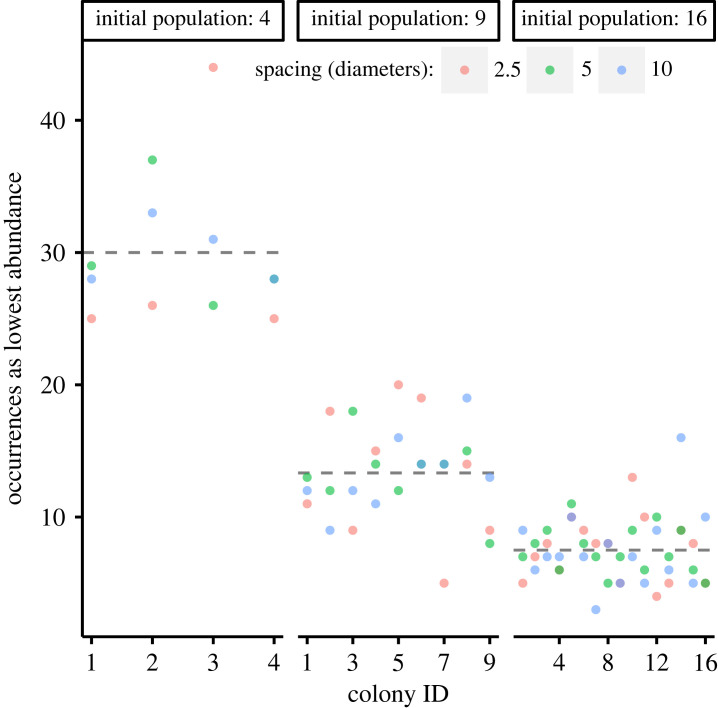
The number of times each colony was the least successful performer during all 120 runs of the baseline simulation where all bacteria were identical. Dashed grey lines indicate the expected value. Points are coloured based on spacings between initial sites. For each set of initial populations, no colony appeared biased away from the expected number of failures.

A foundational assumption of this approach is that even in a system with equally spaced, identical microbes, random growth will lead to drift. We tested this assumption for crowding scenarios where all microbes had identical base *K_s_* and *µ*_max_ parameters by determining the number of times each lineage was the ‘biggest loser’ over 120 simulations (figure 3) and, similar to testing *m* dice for fairness, applied a chi-square test (*α* = 0.05/*m*) where *m* is a Bonferroni correction for multiple testing (*m* = 9 at 3 × 3 initial spacings and population sizes). Each initial site was statistically as likely as any other to be the biggest loser (electronic supplementary material, table S6).

Additionally, the relative proportion of lineages which languished, survived or thrived for each set of crowding conditions was determined. Simulations, on average, had between one and two thriving lineages, with the rest languishing (65–75% for four initial sites, 80–88% others), and a few (0–5%) which did not thrive but grew to non-negligible abundance (electronic supplementary material, table S1). When four organisms were initially present, only languishing and thriving lineages existed, there was otherwise no clear trend between these ratios and either the number or spacing of initial bacteria.

### 3.2. The least successful lineages could overcome drift with altered kinetics

**Figure 4.**
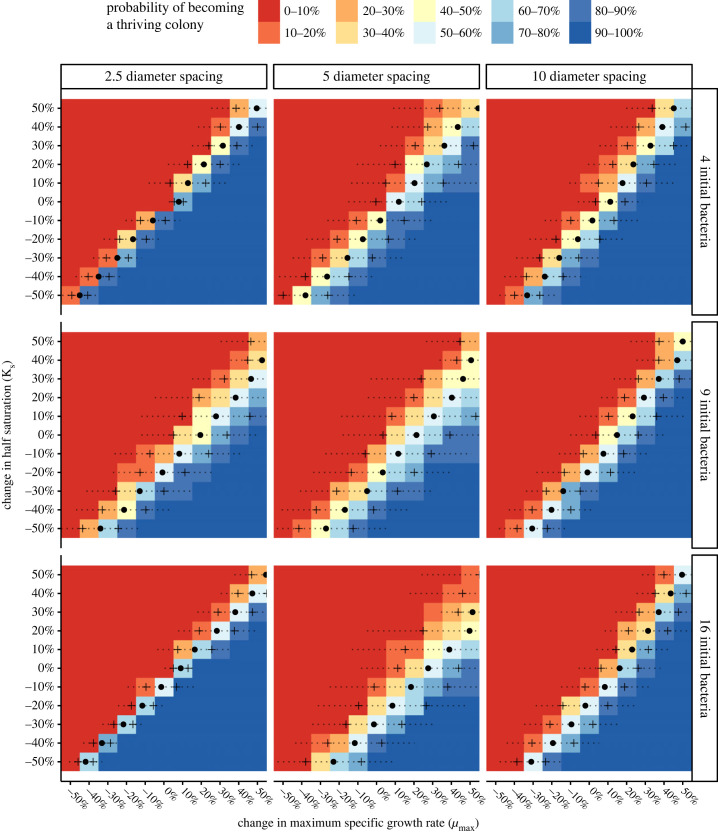
Changing the *µ*_max_ and *K_s_* of the least successful lineage was associated with a probability of transitioning to a thriving status. Solid dots represent *µ_50,_* the per cent change in *µ*_max_ at a given *K_s_* associated with 50–50 odds of thriving_._ Dashed lines show the range of *µ*_max_ corresponding to a *p*_thrive_ of 2.5 to 97.5 (i.e. *spread_95_*). Crosses indicate the analagous *spread_68_* region.

As expected, altering the kinetics of an organism could give it a chance to overcome drift-driven failure (figure 4).

The increases in *µ*_max_ corresponding to the least successful lineage having a 50% chance to become thriving, which we denote as *µ_50_*, are represented by the dark circles in figure 4. At the baseline *K_s_* a typical *µ_50_* is in the range of 10–30%, with the exact value affected by initial spacing and population size (i.e. crowding). Decreasing *K_s_*_,_ as expected, reduces *µ_50_*, even to the point where so long as substrate uptake affinities are ‘good enough’, the initially failing organism may have excellent odds despite having a *µ*_max_ notably lower than its peers. The overall effect, for a given crowding condition, is a semi-linear ‘cliff' of *µ_50_* values where *µ_50_* changes inversely with *K_s_*. Qualitatively speaking, the location of that ‘cliff’ was shifted to the right (higher *µ_50_*) when crowding was increased via initial population size or when comparing between the extremes of spacing.

Areas where the probability of thriving is neither 0 nor 1, are, by definition, areas where drift and selection both influence success. The widths of these areas are denoted as *spread* and are indicated by the dotted horizontal lines and crosses in figure 4. The full length of the line denotes the *spread_95_* area, which is the range of *µ*_max_ for a given *K_S_* which corresponds to a 2.5% to 97.5% chance of thriving. The crosses represent a similar range, *spread_68_*, which corresponds to a 16% to 85% chance of thriving.

Because the *µ_50_* values are also the centre point of the *spread* regions, spread shifted in the same manner as *µ_50_*. However, the actual magnitudes of *spread* did not necessarily follow the same patterns. First, there was no guaranteed symmetry about *K_s._* For example, for nine initial organisms separated by five diameters, the *spread_95_* for *K_s_* of −30% and 30% are visibly different (figure 4, row 2 column 2). Though the asymmetry varied between crowding conditions, it generally manifested as spread widening with increasing *K_s_*. Second, there was no clear monotonic trend with spread values corresponding to crowding. A spacing of five diameters appeared to produce the widest spreads, *ceteris paribus*. Further, there was no clear rule determining which of the two spacing extremes would have a larger *spread.* For example, with four initial bacteria a spacing of 10 diameters resulted in larger spreads than in 2.5 diameters, but the opposite occurred with 16 initial bacteria.

### 3.3. Quantitative effect of crowding on *µ_50_* and s*pread*

The qualitative effects of crowding described in the previous section were quantified via simple linear regression as described in §2.7.2.

**Figure 5.**
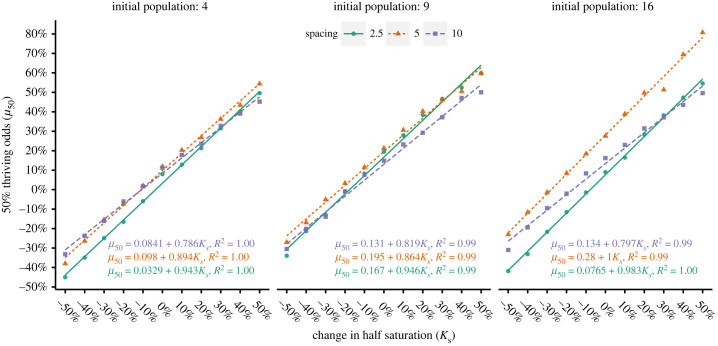
Under each crowding condition, *µ_50_* changed linearly with *K_S_.* Large initial population sizes increased the differences between spacings, moderate spacings generally required the largest absolute *µ_50_*, but the tightest spacings required the largest change *µ_50_* in per unit change in *K_S_*.

For any given crowding condition *µ_50_,* the relative change of *µ*_max_ at which the worst-performing lineage had a 50% chance to transition towards thriving, was essentially linear with respect to *K_S_* and the correlation coefficient was uniformly high (figure 5). The slopes of these relationships indicate the change in *µ_50_* required to compensate for a change in *Ks.* At the tightest spacing, *µ_50_* had to change the most, with a ratio of essentially 1 : 1 and a slight monotonic increase corresponding to initial population size. As initial spacings widened, the ratio almost always decreased for any initial population size. Across initial population sizes, the ratio for 5 and 10 diameter spacings appeared to follow a general trend of increasing, but this was not monotonic.

The absolute value of *µ_50_* was strongly affected by differences between the fitted intercepts. For example, a 2.5 diameter spacing under an initial population size of 16 had a high slope (0.983) but also the lowest required *µ_50_* of all spacings under the same conditions until a 30% change in *K_s_*. The practical difference between spacing was largest at high initial population size, indicating a potential interaction between these factors.

**Figure 6.**
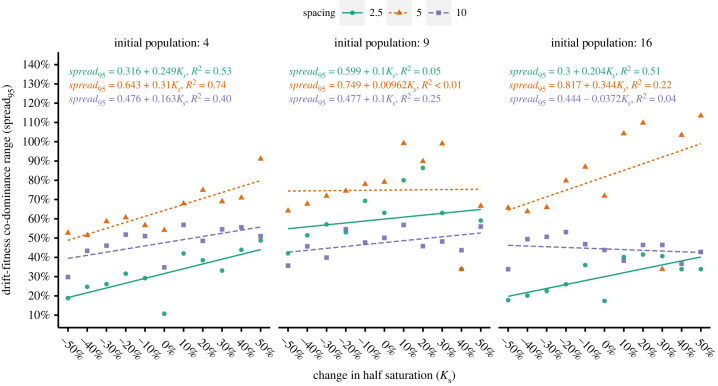
Under each crowding condition, *spread_95_* changed with *K_S_.* Insofar as trends were present, moderate spacing produced the widest *spread_95_* and the differences between spacings increased with population size.

Unlike *µ_50_*, the range over which both drift and selection effects influenced success, *spread_95_* did not have a simple linear relationship with *K_S,_* with many poor *R*^2^ values, residual patterns and high-leverage datapoints (figure 6). There was also no clear, consistent relationship applicable across factors. In general, linear fits became worse with increasing population size which appeared to produce higher variance and generated more high-leverage points, especially at separation distances of five diameters. These issues were largely the same when the analysis was repeated for *spread_68_* (electronic supplementary material, figure S13). There is little to concretely say except that the *spread* was most often widest at moderate spacings, generally increased with *K_s_,* and had a noisy, complicated relationship with initial population size and spacing.

The simulation results were modelled using both MLR and a GAM, respectively, described by equations (3.1) and (3.2) where *p*_thrive_ is the probability of transitioning to a thriving status, *µ_p_* and *K_p_* are the respective per cent changes from the baseline *µ*_max_ and *K_s_*, *N_0_* is the initial population size, *s_i_* is the initial spacing (in diameters) between organisms and *ε* is a small pseudo-probability (1 × 10^−6^) added to avoid division by 0 and issues with log transformation. For linear terms in equations (3.1) and (3.2), *β_i_* denotes the fitted coefficient for term *i* with *i* = 0 representing the intercept. Terms to which GAM smoothing was applied are represented by s(*…*) in equation (3.2) with interactions between a smoothed variable *x* and linear variable *y* denoted as s*(x,* by *y)*. Significant terms (*p* < 0.05) are highlighted in bold. The associated coefficients, significance values and other relevant fitting information are included in the electronic supplementary material, tables S2–S5.

3.1 $$\begin{array}{rl} \log\left(\frac{p_{\mathrm{thrive}}}{1-p_{\mathrm{thrive}}+\varepsilon }+\varepsilon \right) & =\beta_{0}+\beta_{1}\mu_{p}+\beta_{2}K_{p}+\beta_{3}N_{0}\\ & \;+\;\beta_{4}s_{i}+\;\beta_{5}\mu_{p}s_{i}\\ & \;+\beta_{6}K_{p}s_{i}, \end{array}$$

and

3.2 $$\begin{array}{rl} p_{\mathrm{thrive}}= & \beta_{0}+s(\mu_{p})+s(K_{p})+s(N_{0})+s(\;s_{i})+s(\mu_{p}K_{p})+s(\mu_{p}s_{i})\\ & +s(K_{p}s_{i})+s(N_{0},\;by\;s_{i})+s(\mu_{p}K_{p}N_{0})+s(\mu_{p}K_{p}s_{i}). \end{array}$$

**Figure 7.**
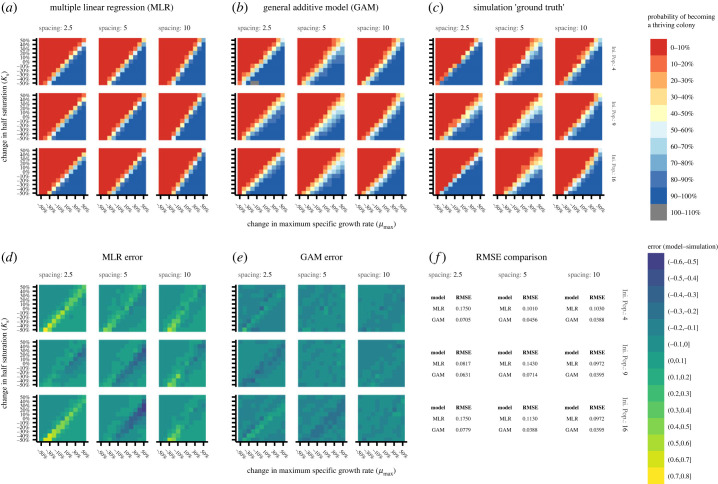
Predictions of MLR model (*a*) and GAM (*b*). Simulation results in (*c*) are presented for ease of comparison. The model errors for the MLR (*d*) and GAM (*e*) are presented visually as well as quantified per-crowding condition in (*f*). The GAM outperformed the MLR, which particularly failed to capture *spread*, was overly optimistic at spacing extremes, and pessimistic at moderate spacing. The small region of greater than 100% odds occurred because the GAM was not constrained to predicting values in the range of [0,1]. Larger individual plots of (*a*), (*b*), (*d*) and (*e*) are available in the electronic supplementary material, figures S14–S17.

The MLR model captured the general behaviour of the shift in the boundary between low and high thriving probabilities but did not adequately reproduce changes in *spread* (figure 7*a* versus figure 7*c*). The overall root-mean-squared error (RMSE) of the model was 0.125. While most predicted probabilities differed from the simulation by no more than ±0.1, some predictions were subject to large error (figure 7*a,d,f*; electronic supplementary material, figures S11, S14 and S15). The largest errors unsurprisingly appear closest to the boundary between low and high *p*_thrive_ regions with the MLR model over-optimistic at the extremes of spacing and lower initial population size. Conversely, the model tended towards overly pessimistic at moderate spacing.

In comparison to the MLR model, the GAM not only captured the general boundary shift but also the changes in *spread* (figure 7*b* versus figure 7*c* in contrast with figure 7*a* versus figure 7*c*). The overall RMSE of the GAM was 0.0563, or somewhat better than half the RMSE of the MLR model. As with the MLR model, most predicted probabilities differed from the simulation by no more than ±0.1. Unlike the MLR model, there were fewer exceptionally large errors and those which did occur were of smaller magnitude (figure 7*b,e,f*; electronic supplementary material, figures S12, S16 and S17). The GAM followed the same trends in over- and under-prediction as the MLR.

## 4. Discussion

### 4.1. Crowding affects the balance between drift and selection

The two parameters describing the balance between drift and selection, *µ_50_* and *spread*, were both affected as crowding became more intense due to either decreased initial spacing or increased initial population size. It was originally expected that as crowding intensity increased, greater selective advantages would be required (*µ_50_*) along with a decrease in the range of values over which both drift and selection influenced success (*spread*). That was not the case.

Instead, the largest *spread* values predominately occurred at moderate (five diameter) initial spacing. We suggest the cause is physical competition for space, specifically the practical significance of single ‘bad’ random choices in division direction and biomass allocation. When bunched tightly together, competition for space is intense and even a few poor random events can consign a lineage to languishing despite a moderate growth advantage. At the other extreme, spatial competition is lessened sufficiently that a few missteps do not guarantee ruin, allowing a lineage to take the full benefit of any growth advantage. Meanwhile, at moderate spacing, immediate neighbours are close enough so that poor random events are harmful but not necessarily disastrous and, at the same time, growth advantages are somewhat hindered, but still helpful. Remembering that spread quantifies the region where both fitness and drift influenced success, it then makes sense that we observed the largest spread values at moderate spacing.

The 50–50 odds point, *µ_50_*, was also slightly larger at moderate spacings, although not consistently and the effect size was not practically different except at large population sizes. The underlying basis for why is not entirely clear, numerically it was due to the consistently larger intercept (figure 5). The trend of the slopes is, however, more easily explained and we attribute it to competition for substrate. For any initial population size, smaller spacings resulted in higher slopes. In other words, to maintain the 50–50 odds when *K_s_* was poor*, µ_50_* had to change more at closer spacing. This makes intuitive sense—closer spacings result in lower local substrate concentrations, and any deficit to *K_s_* is more deleterious to selection.

Increased initial population sizes had more straightforward, secondary, effects on *µ_50_* and *K_s_*. As the initial population size increased, the differences between spacings became more pronounced, but the general trends remained unchanged. In other words, more competitors are problematic, especially as it relates to diffusible substrate, but the major influence on success is competition for space between immediate neighbours.

### 4.2. Interactions between factors incorporating nonlinear effects are important

In the MLR, a main effects only model (RMSE 0.125, *R*^2^ of 0.820) performed essentially identically to the MLR model with interactions (RMSE 0.127, and *R*^2^ of 0.820); however, neither adequately reproduced simulation results. Both were especially poor at representing the regions where fitness and drift influenced success. A GAM which incorporated only main effects using nonlinear smoothing quantitatively performed slightly worse than either MLR main effects model (RMSE 0.197 and *R*^2^ of 78.1), but drastically and uniformly overpredicted spread. Only when both interactions and smoothing were incorporated did a model adequately reproduce the simulation results (figure 7; electronic supplementary material, figure S17). It is visually apparent in the simulation results and quantified in the fitting results (electronic supplementary material, tables S4 and S5) that interactions are important, particularly those involving spacing. Further, the nonlinearity of the interactions (measured as the departure of the term's extended degrees of freedom from a value of 1) is particularly high for any interaction incorporating both *µ_p_* and *K_p_* and less so but still notably for interactions incorporating spacing (electronic supplementary material, table S5).

### 4.3. Limitations and extensions

The simulated conditions were deliberately chosen to isolate the effect of drift. While this made the work tractable, a system wherein every organism is completely identical, starts growing at the same time and is initially evenly spaced on a grid, does not frequently occur in nature. Although we believe the general themes uncovered translate to real ecological systems, the exact quantification does not and is not meant to apply to all situations. Future work should focus on stochastically placed (in time and space) populations with natural variability in Monod parameters.

Extending the work so that the simulated community reflects a more natural distribution would also enable validation of the model, as, despite promising advances [46], it is currently infeasible to exactly place essentially identical bacteria at the resolution required.

Additional parameters affecting drift and selection should also be evaluated—especially the influence of nutrient-rich conditions [47] and how a change to yield, rather than growth rate, alters success [48]. Adding these factors requires, however, overcoming the curse of dimensionality, the current simulations took over 1 year of real-world time and 175 years’ worth of CPU time. Given the large areas where ‘nothing interesting’ happens, designing further experiments to incorporate adaptive sampling [49] is a promising solution. Further, adaptive sampling would enable, at the same computational cost, exploring a larger range of *µ*_max_ and *K_S_* variation (which may vary by orders of magnitude in real-world conditions [50]) and at a greater degree of resolution than 10% changes in the region where the probabilities rapidly change.

## 5. Conclusion and relevance to real-world systems

It is apparent that during biofilm formation in low-nutrient conditions, drift strongly determines which organisms thrive and which organisms fail, so long as they have similar growth rates and substrate affinities. Even when those parameters differ between individuals by ±50%, there are still large regions where a selective advantage does not guarantee overcoming drift-driven failure.

In fact, we observed the lineage fates were determined very early in the simulations and for these systems ‘well-begun is half done’. We speculate that this may be a piece of the puzzle explaining the apparent contradiction between actual and effective community size in neutral modelling [4]—the bacteria are not in competition with the full steady state community but only the immediate smaller community near the beginning of biofilm growth. However, the conditions studied here violate the steady state assumption of that work, so a more careful analysis is warranted.

The conditions we have described are not dissimilar from those within an aerated portion of a wastewater treatment plant, where tightly packed bacterial aggregates are suspended in a bulk liquid and where substrate concentrations are often quite low, especially during operation as a completely mixed stirred reactor (albeit somewhat higher than simulated here). Further, these bacteria are recirculated through the system and relatively well-adapted to domestic wastewater, thus already selected for similarity. Based on the results presented here, we would expect to see a system in which there is a high degree of random turnover in organism identity, but relatively stable functional and biological activity, which is exactly what has been observed in wastewater treatment plants [51,52].

## Data Availability

The data analysis code, data from the simulations and exact NUFEB variant are, respectively, located in the following repositories: Analysis: https://github.com/joeweaver/agent_based_biofilm_drift. Data: https://osf.io/fch3z/. NUFEB variant: https://github.com/nufeb/NUFEB-dev/tree/compute_vol_group The data are provided in the electronic supplementary material [53].

## References

[1] Vellend M. 2010 Conceptual synthesis in community ecology. Q. Rev. Biol. **85**, 183-206. (10.1086/652373) 20565040

[2] Battin TJ, Sloan WT, Kjelleberg S, Daims H, Head IM, Curtis TP, Eberl L. 2007 Microbial landscapes: new paths to biofilm research. Nat. Rev. Microbiol. **5**, 76-81. (10.1038/nrmicro1556) 17170748

[3] Nemergut DR et al. 2013 Patterns and processes of microbial community assembly. Microbiol. Mol. Biol. Rev. **77**, 342-356. (10.1128/MMBR.00051-12) 24006468 PMC3811611

[4] Sloan WT, Nnaji CF, Lunn M, Curtis TP, Colloms SD, Couto JM, Pinto AJ, Connelly S, Rosser SJ. 2021 Drift dynamics in microbial communities and the effective community size. Environ. Microbiol. **23**, 2473-2483. (10.1111/1462-2920.15453) 33684262

[5] Zhou J et al. 2013 Stochastic assembly leads to alternative communities with distinct functions in a bioreactor microbial community. MBIO **4**, e00584-12. (10.1128/mBio.00584-12) 23462114 PMC3585448

[6] Dini-Andreote F, Stegen JC, van Elsas JD, Salles JF. 2015 Disentangling mechanisms that mediate the balance between stochastic and deterministic processes in microbial succession. Proc. Natl Acad. Sci. USA **112**, E1326-E1332. (10.1073/pnas.1414261112) 25733885 PMC4371938

[7] Cira NJ, Pearce MT, Quake SR. 2018 Neutral and selective dynamics in a synthetic microbial community. Proc. Natl Acad. Sci. USA **115**, E9842-8. (10.1073/pnas.1808118115) 30266791 PMC6196497

[8] Fodelianakis S, Valenzuela-Cuevas A, Barozzi A, Daffonchio D. 2021 Direct quantification of ecological drift at the population level in synthetic bacterial communities. ISME J. **15**, 55-66. (10.1038/s41396-020-00754-4) 32855435 PMC7852547

[9] Li B et al. 2019 NuFeb: a massively parallel simulator for individual-based modelling of microbial communities. PLoS Comput. Biol. **15**, e1007125. (10.1371/journal.pcbi.1007125) 31830032 PMC6932830

[10] Jayathilake PG et al. 2017 A mechanistic individual-based model of microbial communities. PLoS ONE **12**, e0181965. (10.1371/journal.pone.0181965) 28771505 PMC5542553

[11] Jayathilake PG, Jana S, Rushton S, Swailes D, Bridgens B, Curtis T, Chen J. 2017 Extracellular polymeric substance production and aggregated bacteria colonization influence the competition of microbes in biofilms. Front. Microbiol. **8**, 1865. (10.3389/fmicb.2017.01865) 29021783 PMC5623813

[12] Sakkos JK, Santos-Merino M, Kokarakis EJ, Li B, Fuentes-Cabrera M, Zuliani P, Ducat DC. 2022 Predicting partner fitness based on spatial structuring in a light-driven microbial community. *bioRxiv,* 2022.09.28.510001. (10.1101/2022.09.28.510001) PMC10184905 37134119

[13] Mölder F et al. 2021 Sustainable data analysis with snakemake. F1000Research **10**, 1-29. (10.12688/f1000research.29032.2) 34035898 PMC8114187

[14] Köster J, Rahmann S. 2012 Snakemake—a scalable bioinformatics workflow engine. Bioinformatics **28**, 2520-2522. (10.1093/bioinformatics/bts480) 22908215

[15] Jette M, Yoo A, Grondona M.2003 SLURM: Simple Linux Utility for Resource Management. In Job scheduling strategies for parallel processing. Lecture Notes in Computer Science, vol. 2862 (eds D Feitelson, L Rudolph, U Schwiegelshohn). Berlin, Germany: Springer.

[16] Ramey C. 1994 Bash, the bourne-again shell. In Proc. of The Romanian Open Systems Conf. & Exhibition (ROSE 1994), pp. 3-5. Bucharest, Romania: The Romanian UNIX User's Group (GURU).

[17] Van Rossum G, Drake FL. 2009 Python 3 reference manual. Scotts Valley, CA: CreateSpace.

[18] Harris CR et al. 2020 Array programming with NumPy. Nature **585**, 357-362. (10.1038/s41586-020-2649-2) 32939066 PMC7759461

[19] McKinney W. 2010 Data structures for statistical computing in Python. In Proc. of the 9th Python in Science Conf., Austin, TX, 28 June–3 July (eds S Walt, J van der Millman), pp. 56-61. See https://conference.scipy.org/proceedings/scipy2010/mckinney.html.

[20] R Core Team. 2022 R: a language and environment for statistical computing. Vienna, Austria: R Foundation for Statistical Computing.

[21] Wickham H et al. 2019 Welcome to the Tidyverse. J. Open Source Softw. **4**, 1686. (10.21105/joss.01686)

[22] Aphalo PJ. 2022 *Ggpmisc: Miscellaneous Extensions to “Ggplot2”*. https://CRAN.R-project.org/package=ggpmisc.

[23] Aphalo PJ. 2022 *Ggpp: Grammar Extensions to “Ggplot2”*. https://CRAN.R-project.org/package=ggpp.

[24] van den Brand, T. 2022 *Ggh4x: Hacks for “Ggplot2”*. https://CRAN.R-project.org/package=ggh4x.

[25] Coretta S. 2022 *Tidymv: Tidy Model Visualisation for Generalised Additive Models*. https://CRAN.R-project.org/package=tidymv.

[26] Daróczi G. 2021 *Logger: A Lightweight, Modern and Flexible Logging Utility*. https://CRAN.R-project.org/package=logger.

[27] Garnier S, Ross N, Rudis R, Camargo P, Sciaini A, Scherer C. 2021 Viridis—colorblind-friendly color maps for R. See https://sjmgarnier.github.io/viridis/. (10.5281/zenodo.4679424)

[28] Henry L, Wickham, H. 2020 *Purrr: Functional Programming Tools*. https://CRAN.R-project.org/package=purrr.

[29] Iannone R, Cheng J, Schloerke B. 2022 *Gt: Easily Create Presentation-Ready Display Table*. https://CRAN.R-project.org/package=gt.

[30] Kassambara A. 2020 *Ggpubr: “ggplot2” Based Publication Ready Plots*. https://CRAN.R-project.org/package=ggpubr.

[31] Meschiari S. 2022 *Latex2exp: Use LaTeX Expressions in Plots*. https://CRAN.R-project.org/package=latex2exp.

[32] Müller K. 2020 *Here: A Simpler Way to Find Your Files*. https://CRAN.R-project.org/package=here.

[33] Neuwirth E. 2022 *RColorBrewer: ColorBrewer Palettes*. https://CRAN.R-project.org/package=RColorBrewer.

[34] Pedersen TL. 2020 *Patchwork: The Composer of Plots*. https://CRAN.R-project.org/package=patchwork.

[35] van Rij J, Wieling M, Baayen RH, van Rijn H. 2022 *Itsadug: Interpreting Time Series and Autocorrelated Data Using GAMMs*. https://CRAN.R-project.org/package=itsadug.

[36] Wickham H. 2016 Ggplot2: elegant graphics for data analysis. New York, NY: Springer-Verlag.

[37] Wilke CO. 2020 *Cowplot: Streamlined Plot Theme and Plot Annotations for “Ggplot2”*. https://CRAN.R-project.org/package=cowplot.

[38] Wilke CO. 2020 *Ggtext: Improved Text Rendering Support for “Ggplot2”*. https://CRAN.R-project.org/package=ggtext.

[39] Wolen AR, Hartgerink CHJ, Hafen R, Richards BG, Soderberg CK, York TP. 2020 Osfr: an R interface to the open science framework. J. Open Source Softw. **5**, 2071. (10.21105/joss.02071)

[40] Wood SN. 2011 Fast stable restricted maximum likelihood and marginal likelihood estimation of semiparametric generalized linear models. J. R. Stat. Soc. **73**, 3-36. (10.1111/j.1467-9868.2010.00749.x)

[41] Pedersen TL, Robinson D. 2022 *Gganimate: A Grammar of Animated Graphics*. https://CRAN.R-project.org/package=gganimate.

[42] Pedersen TL. 2022 *Transformr: Polygon and Path Transformations*. https://CRAN.R-project.org/package=transformr.

[43] Ooms J. 2022 *Gifski:* *Highest Quality GIF Encoder*. https://CRAN.R-project.org/package=gifski.

[44] Caswell H. 1976 The validation problem. Syst. Anal. Simul. Ecol. **4**, 313-325. (10.1016/B978-0-12-547204-3.50050-6)

[45] Hastie T, Tibshirani R. 1987 Generalized additive models: some applications. J. Am.Stat. Assoc. **82**, 371-386. (10.1080/01621459.1987.10478440)

[46] Krishna Kumar R et al. 2021 Droplet printing reveals the importance of micron-scale structure for bacterial ecology. Nat. Commun. **12**, 857. (10.1038/s41467-021-20996-w) 33558498 PMC7870943

[47] Nadell CD, Foster KR, Xavier JB. 2010 Emergence of spatial structure in cell groups and the evolution of cooperation. PLoS Comput. Biol. **6**, e1000716. (10.1371/journal.pcbi.1000716) 20333237 PMC2841614

[48] Kreft JU, Bonhoeffer S. 2005 The evolution of groups of cooperating bacteria and the growth rate versus yield trade-off. Microbiology **151**, 637-641. (10.1099/mic.0.27415-0) 15758209

[49] Westermann PW, Evins R. 2019 Adaptive sampling for building simulation surrogate model derivation using the LOLA-Voronoi algorithm. Proc. Int. Building Performance Simul. Assoc. **16**, 1559-1563.

[50] Cox CD. 2004 Statistical distributions of uncertainty and variability in activated sludge model parameters. Water Environ. Res. **76**, 2672-2685. (10.1002/j.1554-7531.2004.tb00229.x) 16042115

[51] Weaver JE. 2021 From floc to reactor scales: a multi-faceted investigation integrating microbial ecological experiments and computational modeling to understand aerobic wastewater systems. Raleigh, NC: North Carolina State University.

[52] Wang X, Wen X, Criddle C, Yan H, Zhang Y, Ding K. 2010 Bacterial community dynamics in two full-scale wastewater treatment systems with functional stability. J. Appl. Microbiol. **109**, 1218-1226. (10.1111/j.1365-2672.2010.04742.x) 20477893

[53] Weaver JE. 2023 Quantifying drift-selection balance using an agent-based biofilm model of identical heterotrophs under low-nutrient conditions. Figshare. (10.6084/m9.figshare.c.6501942) PMC10251119 37303747

